# Adhesion of Gastric Cancer Cells to the Enteric Nervous System: Comparison between the Intestinal Type and Diffuse Type of Gastric Cancer

**DOI:** 10.3390/cancers14143296

**Published:** 2022-07-06

**Authors:** Paul Girot, Nicolas Chapelle, Laetitia Aymeric, Anne Bessard, Alice Prigent, Yann Touchefeu, Christine Varon, Michel Neunlist, Emilie Duchalais, Tamara Matysiak-Budnik

**Affiliations:** 1 The Enteric Nervous System in Gut and Brain Disorders, Université de Nantes, INSERM, TENS, IMAD, F-44000 Nantes, France; paul.girot@chd-vendee.fr (P.G.); nicolas.chapelle@chu-nantes.fr (N.C.); laetitia.aymeric@univ-nantes.fr (L.A.); anne.bessard@univ-nantes.fr (A.B.); prigent.alice@gmail.com (A.P.); yann.touchefeu@chu-nantes.fr (Y.T.); michel.neunlist@univ-nantes.fr (M.N.); emilie.duchalais@chu-nantes.fr (E.D.); 2 Hepato-Gastroenterology & Digestive Oncology, University Hospital of Nantes, 1 Place Alexis Ricordeau, F-44093 Nantes, France; 3 Department of Biology, Université d’Angers, F-44045 Angers, France; 4 INSERM, U1053, Université de Bordeaux, F-33000 Bordeaux, France; christine.varon@u-bordeaux.fr

**Keywords:** gastric cancer, enteric nervous system, cell-adhesion molecules, diffuse type, intestinal type

## Abstract

**Simple Summary:**

Gastric cancer is one of the leading cause of cancer-related deaths worldwide. The role of the enteric nervous system, a component of the tumor micro-environment in digestive cancers is of growing interest, since enteric neurons may be a route for cancer dissemination. The aim of this study was to investigate the adhesion of the gastric cancer cells to the neurons of the enteric nervous system. We showed that enteric neurons were a privileged site of gastric cancer cell adhesion, particularly in diffuse-type gastric cancer cell lines. We showed that this phenomenon was partially mediated by N-Cadherin, an adhesion protein whose blockade resulted in a dramatic decrease in adhesion ability. These results support the role of enteric neurons as a potential route for cancer cells dissemination, particularly in diffuse-type gastric cancer.

**Abstract:**

Background: Gastric cancer (GC) is the third leading cause of cancer-related deaths worldwide. The enteric nervous system (ENS) has been suggested to be involved in cancer development and spread. Objective: To analyze the GC cell adhesion to the ENS in a model of co-culture of gastric ENS with GC cells. Methods: Primary culture of gastric ENS (pcgENS), derived from a rat embryo stomach, was developed. The adhesion of GC cells to pcgENS was studied using a co-culture model. The role of N-Cadherin, a cell-adhesion protein, was evaluated. Results: Compared to intestinal-type GC cells, the diffuse-type GC cancer cells showed higher adhesion to pcgENS (55.9% ± 1.075 vs. 38.9% ± 0.6611, respectively, *p* < 0.001). The number of diffuse-type GC cells adherent to pcgENS was significantly lower in neuron-free pcgENS compared to neuron-containing pcgENS (*p* = 0.0261 and 0.0329 for AGS and MKN45, respectively). Confocal microscopy showed that GC cells adhere preferentially to the neurons of the pcgENS. N-Cadherin blockage resulted in significantly decreased adhesion of the GC cells to the pcgENS (*p* < 0.01). Conclusion: These results suggest a potential role of enteric neurons in the dissemination of GC cells, especially of the diffuse-type, partly through N-Cadherin.

## 1. Introduction 

Gastric cancer (GC) is the fifth most common cancer and the third leading cause of cancer-related deaths in the world [1]. Although its incidence has been decreasing in most countries in the last decades, some recent epidemiological studies show its rising incidence, especially in younger patients [2]. GC is a heterogeneous disease, and according to its histological characteristics, two main types of GC are distinguished by the Lauren classification [3]: the intestinal type, whose incidence is decreasing and which affects mainly elderly patients, and the diffuse type, which is becoming more frequent and affects younger patients. Diffuse-type GC is considered a negative prognostic factor compared to intestinal-type histology [4,5]. Diffuse-type GC also shows a particular dissemination pattern, with a tropism for lymph nodes and a tendency for intra-peritoneal dissemination rather than metastatic spread to solid organs (liver, lung), which is typical for the intestinal type [6]. The mechanisms underlying these different patterns of evolution remain largely unknown.

Over the last decades, the tumor microenvironment has been progressively recognized as a key contributor to tumor growth and distant dissemination through its interactions with tumor cells [7,8]. In addition to the “classic” components of the tumor microenvironment (blood vessels, fibroblasts, immune cells, extracellular matrix), there is growing evidence of an important role played by the nervous system, a newly recognized tumor microenvironment constituent, in both tumor growth and spread. For decades, the importance of the interactions between cancer cells and neuronal structures has been highlighted, and perineural invasion, defined as the presence of cancer cells along the nerves and/or inside the epineurial, perineurial, and endoneurial spaces of the neuronal sheath [9], has been shown to be associated with a poorer prognosis [10]. In GC, perineural invasion is significantly more common in diffuse-type and poorly differentiated GCs, which could in part explain their poor prognostic characteristics [11]. The perineural invasion has also been described as a route for local growth and metastatic dissemination in pancreatic and prostate cancers [12,13]. In colorectal cancer, it has been shown that cancer cells adhere to and migrate along enteric neurons partly via N-cadherin, a protein associated with the epithelial to mesenchymal transition [14], but its role has never been studied in GC.

Therefore, our aim was to study the in vitro adhesion of GC cells to the components of the gastric enteric nervous system (ENS) using a newly developed co-culture model. In particular, we wanted to compare the levels of adhesion between the diffuse type and intestinal type of GC and study whether the cell adhesion molecules previously identified in colorectal cancer are also involved in GC.

## 2. Materials and Methods

### 2.1. Cell Lines

Diffuse-type GC cell lines, AGS and MKN45, and intestinal-type cell lines, MKN74 and NCI87, were used. The AGS and MKN45 cells were cultured in Roswell Park Memorial Institute (RPMI)-1640 medium (Gibco; Life Technologies, Carlsbad, CA, USA) supplemented with 10% fetal calf serum (FCS; Eurobio Ingen, Les Ulis, France), 50 U/mL penicillin and 50 mg/mL streptomycin (PS; Eurobio Ingen, Les Ulis, France). The MKN74 and NCI87 cells were cultured in Dulbecco’s modified Eagle’s medium (DMEM)-F12 (Gibco Life Technologies, Carlsbad, CA, USA) supplemented with 10% FCS and PS. All cell lines were cultured at 37 °C in a humidified chamber with 5% carbon dioxide. Cells were regularly passaged to maintain exponential growth. All cell lines tested negative for Mycoplasma. Cells were infected with a lentiviral vector hPGK-eGFP (gift from B. Lardeux, UMR Inserm 913, Nantes, France) in order to express the green fluorescent protein (GFP).

### 2.2. Primary Cultures of Gastric ENS

Primary cultures of gastric ENS (pcgENS) were obtained using the revised protocol previously developed in our laboratory for primary cultures of intestinal ENS [15,16] and adapted for gastric ENS. Pregnant Sprague–Dawley rats were purchased from Janvier Labs (Le Genest-Saint-Isle, France) and used in compliance with the French institutional guidelines. All procedures were approved by the local institutional animal research committee (Agreement E.44015; Inserm, Nantes, France). Pregnant rats were killed by an overdose of CO_2_. The E15 embryos were then removed, and their stomachs were extracted and finely diced in Hank’s balanced salt solution (HBSS; Biowest, Nuaillé, France). Tissue fragments were collected in 10 mL of DMEM-F12 1:1 medium and digested at 37 °C for 15 min in 0.1% trypsin (Sigma-Aldrich, St. Louis, MO, USA). The trypsin reaction was stopped by adding 20 mL of medium containing 10% FCS and then treated with DNAse I (0.01%; Sigma-Aldrich, St. Louis, MO, USA) for 10 min at 37 °C. After triturating with a 10 mL pipette, cells were centrifuged at 10^5^ g for 10 min. The cells were then counted and seeded at a density of 2.4 × 10^5^ cells/cm^2^ on 24-well plates previously coated with a gelatin solution (0.5%; Sigma-Aldrich, St. Louis, MO, USA) for 6 h in sterile phosphate-buffered saline (PBS). After 24 h, the medium was replaced with a serum-free medium, half Ham F12 nutrient mixture (Gibco; Life Technologies, Carlsbad, CA, USA) and half DMEM without glucose (Gibco; Life Technologies, Carlsbad, CA, USA) containing 1% of N-2 supplement (Life Technologies, Carlsbad, CA, USA). Cells were maintained in culture for 11 days. Half of the medium was replaced twice a week. There was no specific selection of any structures (enteric neurons, glia…) during this process. The composition of the pcgENS was only driven by the culture medium, facilitating ENS growth without supplementary growth or differentiation factors.

### 2.3. Characterization of GC Cell Lines and pcgENS

#### 2.3.1. Immunofluorescence

Cell cultures or pcgENS (at day 11) were fixed with 4% paraformaldehyde in PBS at room temperature for 30 min. After permeabilization with PBS-sodium azide containing 10% horse serum and 0.25% Triton X-100 (Sigma-Aldrich, St. Louis, MO, USA), samples were incubated at room temperature, sequentially, with primary antibodies for 3 h, washed 3 times with PBS, and then with secondary antibodies for 1 h, diluted in PBS-sodium azide containing 10% horse serum. The following primary antibodies and dilutions were used for immunofluorescence microscopy experiments: rabbit anti S100β (1:200, IR504, Dako, Santa Clara, CA, USA) staining glial cells; goat anti α-smooth muscle actin (SMA) (1:200, ab21027; Abcam, Cambridge, UK) staining smooth muscle; mouse anti PGP9.5 (1:500, MA1-83428, Thermo Fisher Scientific, Waltham, MA, USA); rabbit anti PGP9.5 (1:500, CL7755AP, Cedarlane, Burlington, ON, Canada) staining enteric nerves and rabbit anti-N-Cadherin (1:200, Ab-18203, Abcam). The following secondary antibodies were used: anti-mouse Cy3 (1/500, 715-165-151, Jackson ImmunoResearch, West Grove, PA, USA), anti-mouse FP 488 (1/500, 115-545-205, Jackson ImmunoResearch), anti-rabbit Cy3 (1/500, 711-165-152, Jackson ImmunoResearch), and anti-goat AMCA (1:50, 705-155-147, Jackson ImmunoResearch).

#### 2.3.2. Western Blot

The pcgENS or tumor cells were lysed in radio-immuno-precipitation assay buffer (Merck Millipore, Fontenay sous Bois, France), followed by sonication for 1 min with “vibracell 75 186” device (Sonics, Newton, CT, USA). Samples were further prepared for electrophoresis by dilution with the NuPAGE sample buffer (Life Technologies, Saint-Aubin, France), then heated at 98 °C for 5 min. Lysates were separated using the NuPAGE 4–12% Bis-Tris gels (Life Technologies) together with the 2-(N-morpholino) ethane sulfonic acid/sodium dodecyl sulfate running buffer (Life Technologies) before electrophoretic transfer to nitrocellulose membranes (Life Technologies) with the iBlotTM Dry Blotting System (Thermofisher Scientific). Membranes were blocked for 1 h at room temperature in Tris-buffered saline (Sigma) with 0.1% (*v*/*v*) Tween-20 (Sigma) and 5% (*v*/*v*) non-fat dry milk or bovine serum albumin (Sigma, A730-500G) and incubated overnight at 4 °C with the following primary antibodies: rabbit anti-N-Cadherin (1:500, Ab 18203, Abcam).

To confirm equal protein loading, membranes were probed with mouse monoclonal anti-β-actin antibody (1:5000, A5441, Sigma). Bound antibodies were detected with horseradish peroxidase-conjugated anti-rabbit (1:5000, 31460, Life Technologies) or anti-mouse antibodies (1:5000, A9044, Sigma-Aldrich) and visualized by enhanced chemiluminescent detection ChemidocTM XRS+ (Biorad, Marnes-la-Coquette, France). To quantify the immunoreactivity of each antibody, the band intensity was analyzed using Image Lab (Biorad, Marnes-la-Coquette, France).

### 2.4. Co-Culture of pcgENS with GFP-Expressing Cell Lines and Treatment with Antibodies

For the GC-cell to ENS adhesion analysis, single-cell suspensions of GFP-expressing diffuse-type (AGS and MKN45) and intestinal-type (MKN74, NCI87) cancer cells were co-cultured with the pcgENS. On day 11, half of the conditioned medium of pcgENS was collected and used to re-suspend GFP GC cells. Non-specific sites of the pcgENS were saturated with bovine serum albumin (Sigma, A730-500G) for 1 h. Then, the cells were co-incubated with pcgENS at a density of 4 × 10^4^ cells/cm^2^ (8 × 10^4^ cells/well) for 1 h. After rinsing with PBS, cultures were fixed for 30 min at room temperature in PBS containing 4% paraformaldehyde (4% PFA), then washed three times in PBS before immunohistochemical staining.

For the experiments with blocking antibodies, after BSA incubation, pcgENS were pretreated with polyclonal mouse IgG (10 µg/mL, ab37355, Abcam, Cambridge, UK) for 1 h and then incubated for 1 h with anti-N-cadherin (clone GC-4, 10 or 20 µg/mL, C3865, Sigma-Aldrich, St. Louis, MO, USA) or polyclonal mouse IgG (10 µg/mL) or media alone. Then, GC cells were added and co-incubated with pcgENS for 1 h, rinsed, and fixed, as previously described.

### 2.5. Microscopy

Conventional microscope imaging of cell cultures was performed using a motorized fluorescence zoom microscope AxioZoom.V16 (Zeiss, Oberkochen, Germany) equipped with an HRm Axiocam (Zeiss). Images were recorded with 1×/0.25 FWD 56 mm objective and processed with Zen software (Zeiss). GC cell to pcgENS adhesion was assessed using confocal microscopy. Images were obtained with an A1 confocal microscope (Nikon, Champigny sur Marne, France), using 20×/0.7 and 60×/1.4 objectives for various magnifications. Images were recorded accordingly to the Nyquist criterion for spatial resolution prior to the deconvolution process (Lucy-Richardson algorithm from Nikon Imaging Software).

### 2.6. Evaluation of GC Cell Adhesion to ENS

For each well, 15 images were taken and analyzed with the image processing program Fiji/ImageJ v1.52b. Then, the mean value for all the measured parameters was calculated for each well. Macro used in Fiji for cell counting, area measurement, and rate of adhered cell measurement are presented in appendix 1. For co-culture analysis, we quantified the proportion of GFP cells located within 1 pixel (<1 µm) of the enteric nerves for each cell line, which were considered adhered cells.

Results of juxtaposed cancer cells were compared between real ENS network pictures (e.g., image 1 of the ENS and image 1 of the GC, same field) to random ENS network images (e.g., image 7 of the ENS and image 1 of the GC, different field), considered as controls. Random ENS network corresponded to the artificial superposition of the image of the pcgENS in a field to the image of GC cells in another field in the well chosen randomly. This method ensures that the superposition/adhesion observed between GC cells and pcgENS was not due to chance.

### 2.7. Statistical Analysis

All data are shown as mean ± standard error of the mean (SEM). The rate of adherent cancer cells, the number of cells, and the area of enteric neurons were compared using the Mann–Whitney test, one-way parametric ANOVA, or Kruskal–Wallis test with Dunn’s post-test when appropriate. Statistical analysis was performed using the Prism software package version 7.04 (Graphpad Prism, La Jolla, CA, USA). A “*p*” value of less than 0.05 was considered statistically significant.

## 3. Results

### 3.1. Gastric Cancer Cells Adhere to the Enteric Neurons of Primary Culture of Gastric ENS in the Co-Culture Model

**Figure 1:**
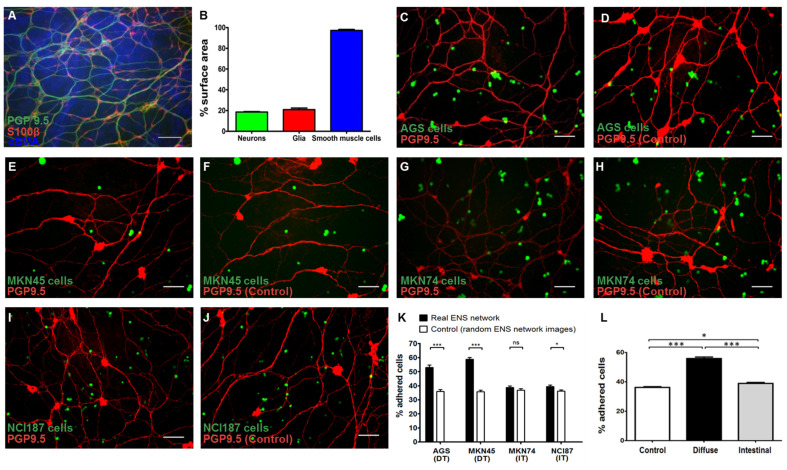
Characterization of the primary culture of gastric enteric nervous system (pcgENS) and adhesion of the GC cells to the enteric nervous structures of the pcgENS. (**A**) Immunostaining of pcgENS on day 11. Staining of pcgENS cellular components using antibodies against PGP9.5 (green), S-100β (red), and α -SMA (blue), illustrating the organization of neurons, glial cells, and smooth muscle cells, respectively. Scale bar: 100 µm. (**B**) Quantification of the area covered by each cellular component present in the primary culture of gastric enteric nervous system (pcgENS): neurons, glial cells (Glia), smooth muscle cells. Data are expressed as mean ± SEM of the percentages relative to the total area of the well (*n* = 10 wells). (**C**–**J**) Enteric neurons (red/PGP9.5) after 1-h incubation with GFP-positive AGS, MKN45, MKN74, and NCI187 cells (green) (**C**,**E**,**G**,**I**, respectively) and the control images merging the corresponding GC cell signal (green) with the enteric neuron signal (red/PGP9.5) from a randomly chosen image (**D**,**F**,**H**,**J** respectively). Scale bar: 100 µm. (**K**) Comparison of the percentage of adhered cells to the percentage of cells which could potentially adhere to a random ENS network image for each cell line (*n* = 19 wells per cell line; ns = non-significant; * *p* < 0.05; *** *p* < 0.001; Mann–Whitney test). (**L**) Comparison of the percentage of adhered cells to neurons between the two types of GC cells (*n* = 38 wells per type). A significantly higher adhesion rate is observed for diffuse-type GC cells (AGS and MKN45 cell lines) compared to the intestinal-type cells (MKN74 and NCI187 cell lines) (* *p* < 0.05; *** *p* < 0.001; one-way parametric ANOVA). DT—diffuse type; IT—intestinal type; ENS—enteric nervous system; pcgENS—primary culture of gastric ENS.

Figure 1 describes the different components of gastric ENS and the relations between GC epithelial cells and the primary culture of gastric ENS in this co-culture model. Immunostaining of pcgENS showed a similar distribution of the components of the gastric ENS when compared to the intestinal ENS, as studied previously [14], which included enteric neurons stained with anti-PGP 9.5 antibodies, covering 18.24% (±0.96%) of the surface; enteric glial cells, stained with anti-S100b, covering 20.88% (±1.46) of the surface; and smooth muscle cells, stained with anti-SMA, covering more than 97.27% (±0.94%) of the surface (Figure 1B) (different components are superposed; therefore, the sum of the surface exceeds 100%).

In the co-culture model, cancer cells covered about 1–2% of the surface of the wells. A superposition of cancer cells and ENS components was observed (Figure 1C–J).

Diffuse-type GC cells adhere more significantly to the enteric nervous structures of the pcgENS than intestinal-type GC cells.

We further investigated the adhesion of the different GC cell lines. Compared to random images, AGS, MKN45, and NCI-87 cell lines adhered significantly more to enteric neurons (Figure 1K). Altogether, diffuse-type [(53.45% (±1.42)] and intestinal-type (38.96% (±0.6611)) cells adhered significantly more to enteric neurons compared to the controls (random images, 36.36% ± 0.4539; *p* < 0.001 and *p* < 0.05, respectively). Interestingly, both AGS and MKN45 cell lines adhered significantly more to ENS structures than MKN74 (*p* < 0.001 and *p* < 0.001, respectively; one-way ANOVA) and NCI cell lines (*p* < 0.001 and *p* < 0.01, respectively; one-way ANOVA) (Figure 1L). Comparison of intestinal-type and diffuse-type GC cells showed that the proportion of cells adherent to the enteric neurons was significantly higher with diffuse-type GC cells (55.91% ± 1.075 vs. 38.96% ± 0.6611; *p* < 0.001). These results further confirm that GC cells, covering between 0.86% and 1.12% of the area in the wells, adhere preferentially to enteric nervous structures covering between 17.71% and 19.08% of the area. Since diffuse-type GC cells adhere significantly more than intestinal-type GC cells, and the difference observed in intestinal-type was less relevant and inconsistent across the different cell lines (not significant, MKN74), we decided to further focus on the diffuse-type.

### 3.2. Enteric Neurons Are Required for Diffuse-Type GC Cell Adhesion to the pcgENS

**Figure 2:**
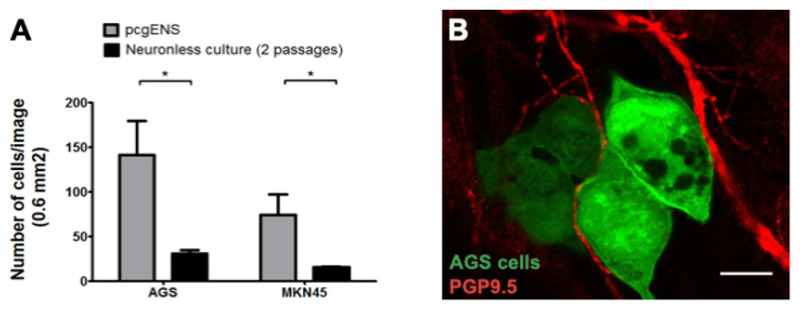
Importance of the enteric neurons in the adhesion of diffuse-type gastric cancer cells to the enteric nervous structures of pcgENS. (**A**) Comparison of cell number per image (0.6 mm^2^) between pcgENS and cultures without neurons after 2 cell passages (*n* = 19 wells for pcgENS and *n* = 10 for P2 cultures; * *p* < 0.05; Mann–Whitney test). (**B**) Confocal imaging of GFP-positive AGS cells adhered to enteric neurons (red/PGP9.5). Scale bar: 10 µm. ENS—enteric nervous system; pcgENS—primary culture of gastric ENS.

To investigate which of the components of the pcgENS was involved in GC cell adhesion, we co-cultured GC cells with neuron-free pcgENS. The neuron-free pcgENS were obtained by applying two cell passages, according to a previously validated protocol, during which the fragile neurons are known to be eliminated (Supplementary Figure S1) [17]. Quantification of the surfaces after immunostaining confirmed the absence of enteric neurons after two passages but the persistence of glial cells (Supplementary Figure S1B). A dramatic reduction in the number of GC cells adherent to the neuron-free pcgENS (mean number of cells/image: AGS: 30.9 ± 4.00, MKN 45:15.69 ± 0.84), compared to enteric neuron-containing pcgENS (mean number cells/image: AGS mean 141.9 ± 38.13; MKN 45 mean 74.7 ± 22.9) was observed (*p* = 0.0261 and *p* = 0.0329 for AGS and MKN 45, respectively) (Figure 2A). These results demonstrate that enteric neurons are required for tumor cell adhesion to the pcgENS.

To investigate whether the superposition observed in the co-culture model corresponded to a true interaction and to assess whether neurons are the adhesion partners of GC cells, confocal microscopy was performed and showed that the superposition corresponded to a true interaction between GC cells and enteric nervous cells (Figure 2B and Figure S2). We thus confirmed the preferential specific adhesion of GC cells to the enteric neurons of the pcgENS in the co-culture model.

### 3.3. Expression of N-Cadherin on Tumor Cells and pcgENS

Our group previously reported that colorectal cancer cells adhered to enteric neurons partly via N-cadherin [14], an adhesion molecule involved in the epithelial-to-mesenchymal transition and overexpressed in colon cancer. To investigate whether this molecule also plays a role in GC, we analyzed N-Cadherin expression in both diffuse-type (AGS, MKN45) and intestinal-type (MKN74, NCI-87) GC cells and in pcgENS, by immunohistochemistry and by Western blot.

**Figure 3:**
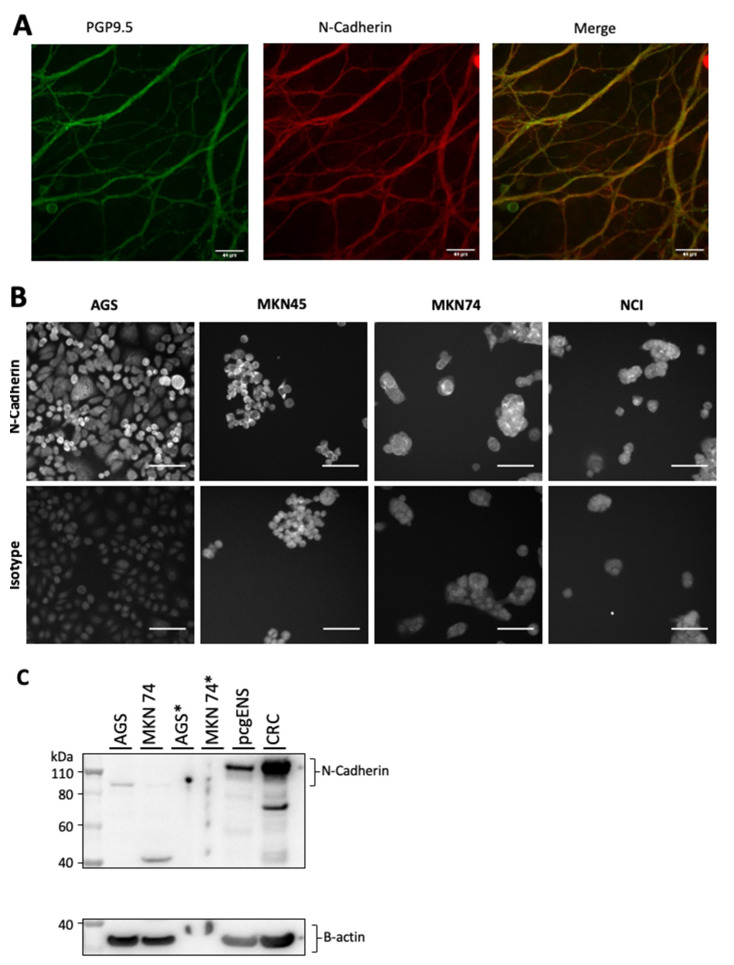
Expression of N-Cadherin in the enteric neurons of the pcgENS and in gastric cancer cell lines. Immunostaining of N-Cadherin using anti-N-cadherin in the enteric neurons of pcgENS (**A**) and in different gastric cancer cell lines (**B**) (scale-bar = 50 µm). Analysis by Western blot of N-Cadherin expression in gastric cancer and CRC cell lines (B-actin used as positive control) (**C**). pcgENS—primary culture of enteric nervous system; CRC—colorectal cancer (human); *—ultracentrifugation protocol. The uncropped Western blots have been shown in Figure S3.

As a major component of the ENS during genesis, not surprisingly, N-Cadherin was found to be expressed by the enteric neurons of pcgENS (Figure 3A).

The expression of N-Cadherin was observed in both diffuse-type cell lines (AGS and MKN45) and intestinal-type cell lines (MKN74 and NCI-87) (Figure 3B).

Western blot showed the presence of N-Cadherin in pcgENS and in a human colorectal cancer sample (positive control) and at lower intensity in AGS and MKN74 cell lines (Figure 3C).

### 3.4. N-Cadherin Is Involved in the Adhesion of GC Cells to Enteric Neurons

**Figure 4:**
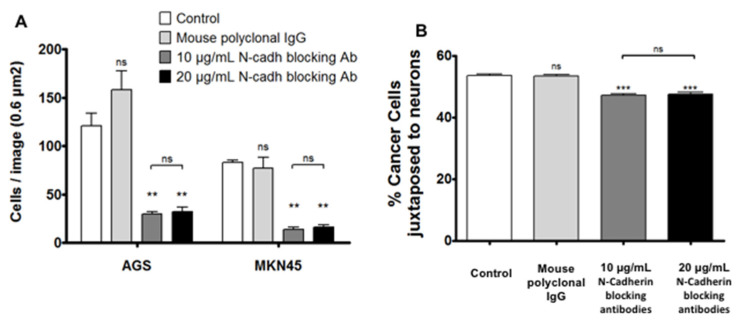
Impact of N-Cadherin on the adhesion of diffuse-type gastric cancer cells to ENS. (**A**) Comparison of the number of cells per image (0.6 mm^2^) between co-cultures of GC cells with pcgENS pretreated for 1 h with anti-N-Cadherin blocking antibody 10 µg/mL, anti-N-Cadherin blocking antibody 20 µg/mL, polyclonal mouse IgG 10 µg/mL, or media alone (*n* = 5 wells per condition; ns = non-significant; ** *p* < 0.01; Ab = antibody; Mann–Whitney test). A significant decrease in tumor cell adhesion of pcgENS is observed after blocking N-Cadherin. (**B**) Comparison of % of adhered cells between co-cultures of GC cells with pcgENS pretreated for 1 h with 10 µg/mL blocking anti-N-Cadherin antibody, 20 µg/mL blocking anti-N-Cadherin antibody, 10 µg/mL polyclonal mouse IgG or media alone (*n* = 10 wells per condition; ns = non-significant; *** *p* < 0.001; Mann–Whitney test).

To further investigate the possible involvement of N-Cadherin in the adhesion phenomenon, we co-cultured diffuse GC cells with pcgENS pre-incubated with N-Cadherin blocking antibodies. The analysis showed that specific N-Cadherin inhibition resulted in a dramatic decrease in adhesion of the tumor cells to the pcgENS: the mean number of cells/image with the medium was 103.9 ± 8.78, with polyclonal IgG 119.3 ± 16.0, with 10 μL/mL N-Cadherin blocking antibodies it was at 22.7 ± 3.12, and with 20 μL/mL N-Cadherin blocking antibodies 25.1 ± 3.6 (*p* = 0.0079) (Figure 4A).

Moreover, this inhibition was significantly related to decreased adhesion to the neurons of the pcgENS: medium 53.5% ± 0.5, polyclonal IgG 53.4% ± 0.7, 10 μL 47.1% ± 0.5, 20 μL/mL 47.4% ± 0.73 (Figure 4B). There was no significant difference between the two concentrations used.

## 4. Discussion

The tumor microenvironment is believed to play an important role in tumor development and spread. Indeed, studies on different components of the tumor microenvironment led to the development of several targeted treatments, such as anti-VEGF, anti-EGFR, or, more recently, anti-PD-1, PD-L1, or CTLA4 antibodies, that considerably improved the prognosis of cancer patients. ENS is of growing interest, and it is considered an important component of the tumor microenvironment in digestive cancers. Recent studies by Zhao et al. and Hayakawa et al. have demonstrated the crucial role of neuronal signals in GC growth and the role of acetylcholine receptors in gastric carcinogenesis [18,19]. We have also previously reported the role of acetylcholine in this process and, in particular, its capacity to induce a stem-cell phenotype of GC cells in an in vitro tumorsphere model [20]. There is also growing evidence that ENS could play a role in tumor dissemination in colorectal cancer [14], but very few data are available for GC.

For the purpose of the present study, we developed an original model of pcgENS co-cultured with GC cell lines, allowing the study of the in vitro relationship between the ENS and GC cells. We first observed the preferential adhesion of GC cells to the ENS and showed that the neurons were required for this adhesion, as demonstrated by a dramatically decreased adhesion in the neuron-free ENS culture. To our knowledge, this is the first report of such an interaction in GC. We also showed that diffuse-type GC cells have higher adhesive properties to neurons compared to the intestinal-type cells. The diffuse type is known to be more aggressive than the intestinal type and shows a particular tropism for enteric neurons, as demonstrated by a higher rate of perineural invasion in clinical studies [11]. A systematic review and meta-analysis showed that perineural invasion in GC is not only associated with decreased overall- and disease-free survival but also with lymph node metastasis [21]. The greater adhesion to neurons may suggest the neuro route as a possible vector for cancer cell dissemination, and several reviews addressed that very particular but poorly investigated pathway for cancer dissemination [22,23]. Our present study suggests that the first step of the process (adhesion) is observed in gastric cancer. Further studies using time-lapse and trajectory analyses are needed to fully validate this hypothesis.

In the previous study focusing on colorectal cancer [14], two adhesion proteins (N-Cadherin and L1-CAM), known markers of the epithelial-to-mesenchymal transition [24], were investigated as potential future targets in cancer treatment. N-Cadherin is part of the cadherin adhesion protein superfamily and is involved in cell migration during nervous system development. Its implication in the polarization, cohesion, and structural organization of the neuroepithelial cells has also been demonstrated [25]. Its expression varies according to the type of cancer and is expressed in about 20% of GC [26] and in about 45% of breast and colon cancers [27,28]. In cancer development, it has been shown that the increased expression of N-Cadherin combined with E-Cadherin silencing (known as “cadherin switching”) was an independent marker of invasiveness and epithelial-to-mesenchymal transition. In the present study, N-Cadherin was found to be expressed by GC cells, and we demonstrated that N-cadherin blocking resulted in a dramatic decrease in cancer cell adhesion to ENS, strongly suggesting that this molecule plays a role in GC cell dissemination via ENS. Although the N-Cadherin expression observed in our study was rather weak, it is possible that it is weakly expressed at the basal level, in particular in vitro, but it may be inducible, notably by GC cell adherence to another structure (in the present case, enteric neurons), as previously described for colorectal cancer [14].

One limitation of the current study is the use of a pcENS derived from another species. The extrapolation of the present findings to humans is questionable. However, to date, no other protocols exist that would allow the culture of enteric structures, and in particular enteric neurons, which are very fragile and difficult to culture from human samples. One solution may be to use induced pluripotent stem cells. However, to our knowledge, this tool does not allow the development of mature enteric neurons, in contrast to the presently used pcENS. Secondly, although the co-culture was carried out using cells from two different species, this does not hamper the interactions, as shown in the present study, as well as in the previous study on colorectal cancer [14]. This may be explained by a highly-conserved structure of N-Cadherin across species.

In melanoma, it has been suggested that heterotypic N-Cadherin adhesion would lead to b-catenin dissociation, favoring the transmigration of cancer cells. It has been suggested that N-Cadherin and the extracellular part of the fibroblast growth factor receptors crosstalk and may play a major role in cancer cell migration [29]. Furthermore, N-Cadherin expression has been extensively investigated in several other cancers (bladder, kidney clear cell, hepatocellular carcinoma, lung, ovarian, cervical squamous cell, and other cancers) and has been recognized as a negative prognostic factor [30]. However, it is, to our knowledge, the first study to demonstrate the direct implication of N-Cadherin in GC, especially of the diffuse type, which could partly explain its higher aggressiveness and neurotropism compared to intestinal-type GC.

## 5. Conclusions

This study, using a newly developed model of co-culture of gastric ENS with GC cells, shows the preferential adhesion of diffuse-type GC cells to the enteric neurons when compared to intestinal-type GC cells. It also shows an inhibitory effect of N-Cadherin blockage on cancer-neuron adhesion, suggesting that perineural invasion could represent a dissemination route for gastric cancer cells.

## Data Availability

Not applicable.

## Supplementary Materials

The following supporting information can be downloaded at: https://www.mdpi.com/article/10.3390/cancers14143296/s1, Figure S1: Characterization of the primary culture of gastric enteric nervous system after 2 passages; Figure S2: Confocal imaging of the adhesion of GC cells to the enteric neurons of the pcgENS; Figure S3: The uncropped Western blots.

## Abbreviations

ANOVA	Analysis of variance
CAM	Cell adhesion molecule
DMEM	Dulbecco’s modified Eagle’s medium
DT	Diffuse type
ENS	Enteric nervous system
FCS	Fetal calf serum
GC	Gastric cancer
GFP	Green fluorescent protein
HBSS	Hank’s balanced salt solution
IT	Intestinal type
PBS	Phosphate-buffered saline
pcgENS	Primary culture of gastric enteric nervous system
PFA	Paraformaldehyde
PNI	Perineural invasion
PS	50 U/mL penicillin and 50 mg/mL streptomycin
RPMI	Roswell Park Memorial Institute
RT	Room temperature
SEM	Standard error to the mean
SMA	α-Smooth muscle actin
TME	Tumor microenvironment
