# Supplementary figures and images for: Adhesion of Gastric Cancer Cells to the Enteric Nervous System: Comparison between the Intestinal Type and Diffuse Type of Gastric Cancer
Source: Cancers (Basel). 2022 Jul 6;14(14):3296. doi: 10.3390/cancers14143296 (PMC9313246; doi:10.3390/cancers14143296)

## Slide 1
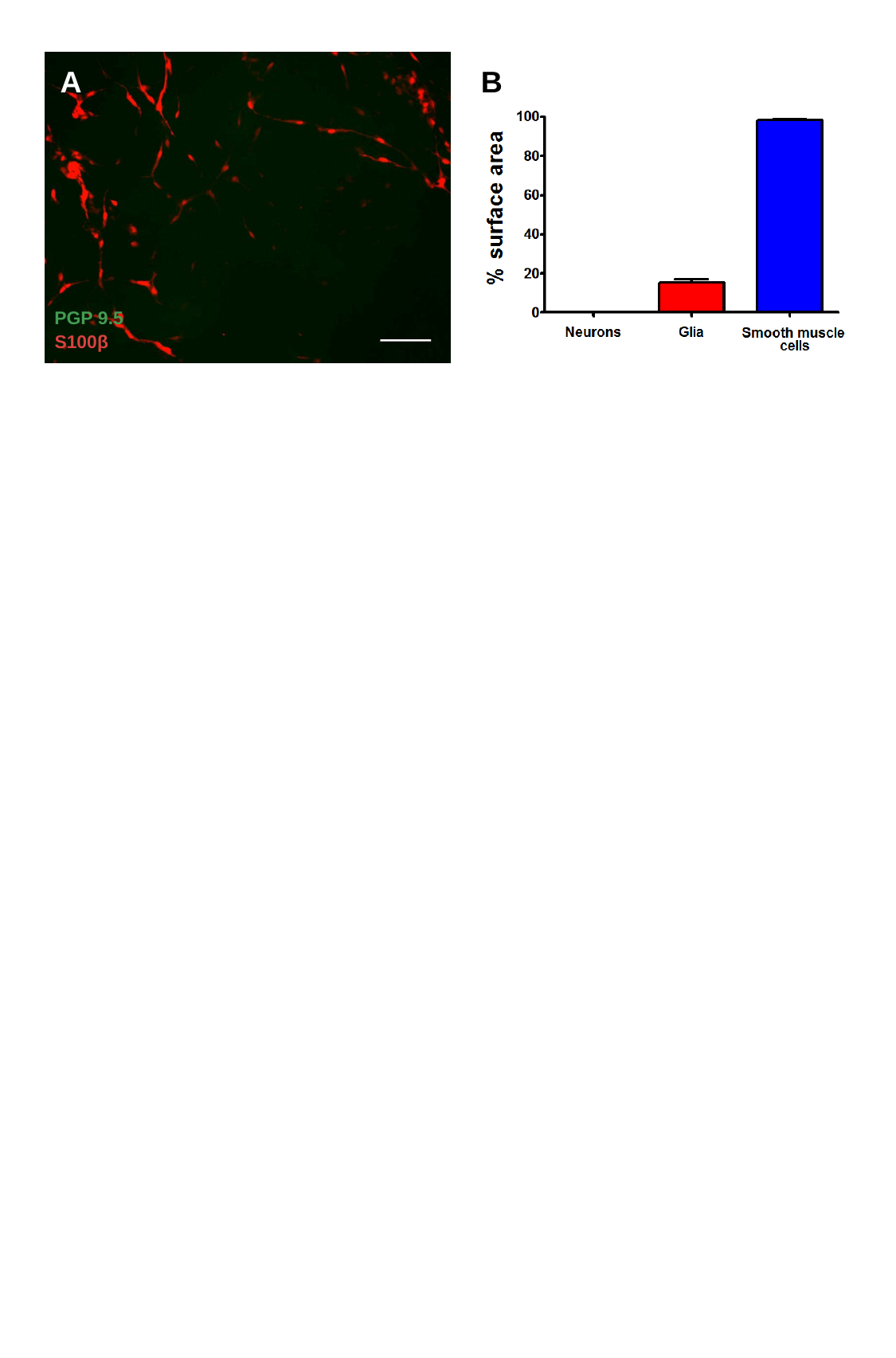

A
B
PGP 9.5
S100β

Supplement: Supplementary file 1 [file cancers-14-03296-s001.zip › Figure S1.pptx]

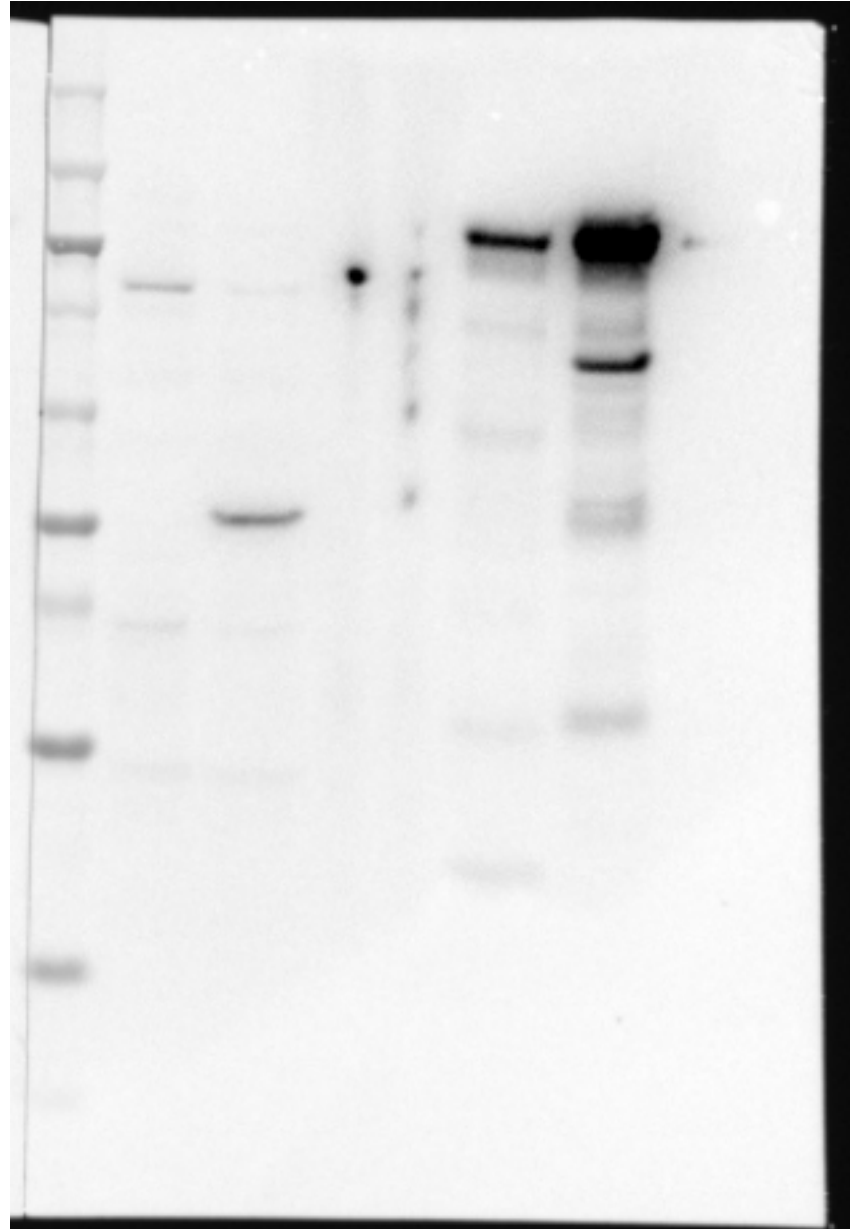

Supplement: Supplementary file 1 [file cancers-14-03296-s001.zip › Figure S3.pdf]
